# Preliminary Study on the Synergistic Degradation Mechanism of the Microbial Community on the Wood of the Dingtao M2 Tomb

**DOI:** 10.3390/ijms27073233

**Published:** 2026-04-02

**Authors:** Cen Wang, Lilong Hou, Yu Wang, Guoming Gao, Yibo Geng, Jiao Pan

**Affiliations:** 1 Key Laboratory of Archaeomaterials and Conservation, Ministry of Education, University of Science and Technology Beijing, Beijing 100083, China; d202410811@xs.ustb.edu.cn (C.W.); d202310766@xs.ustb.edu.cn (Y.W.); m202411471@xs.ustb.edu.cn (G.G.); m202511462@xs.ustb.edu.cn (Y.G.); 2 Institute for Cultural Heritage and History of Science & Technology, University of Science and Technology Beijing, Beijing 100083, China; 3 College of Life Sciences, Nankai University, Tianjin 300071, China; 2120221471@nankai.edu.cn

**Keywords:** Dingtao M2 Tomb, *Penicillium*, synergistic degradation, organic acids, wooden relic conservation

## Abstract

According to our investigation carried out in July 2023, the wood of the Western Han Dynasty Dingtao M2 Tomb, stored in the preservation room, exhibited signs of microbial degradation. Our metagenomic analysis first revealed *Penicillium* as the dominant genus on the end of the wrapped wood. Furthermore, functional annotations demonstrated that the resident microbial community possessed cellulolytic and ligninolytic capabilities. Targeted metabolomic analysis evaluated the degradation capacity of *Penicillium charlesii* DTP_1, a strain isolated from the wrapped wood. We hypothesize that DTP_1 provides an acidic microenvironment via the production of organic acids; the functional microbial community then decomposes lignin into small metabolites via enzymatic action, and these products are then utilized by the microbial community, including DTP_1. Finally, we verified that liquid cinnamaldehyde and volatile gaseous allicin and carvacrol exhibit better inhibitory efficacy. Nevertheless, further optimization of plant-derived agents and application methods are still required. This study proposes a putative mechanism underlying the degradation of the Dingtao M2 Tomb wood by the microbial community, thereby providing theoretical support for the conservation of wooden cultural heritage and relics.

## 1. Introduction

Cultural heritage is the treasure of human civilization, bearing significant value in terms of both tradition and history. However, microbial degradation, a ubiquitous phenomenon worldwide, has emerged as a critical threat to the conservation and preservation of these cultural relics, particularly organic relics such as wooden items and buildings [1,2,3,4]. Microbial degradation may cause serious problems such as decay, deformation, or discoloration, attributed to an abundance of cellulose, hemicellulose, and lignin [5,6], which provide essential nutrients for microbial colonization, growth, and reproduction. Microbes, especially fungi [7], are usually among the main agents which cause the degradation of wooden relics [8,9,10,11]. The growth of fungal hyphae penetrating the cell lumen can cause mechanical damage to cultural relics [10,12]. Meanwhile, enzymes, pigments and other substances secreted by microorganisms can cause irreversible damage to these precious relics [13,14,15]. Filamentous fungi, especially *Penicillium* spp., are commonly found during the microbial degradation of wooden relics [16]. This genus possesses strong capabilities for producing a diverse array of bioactive substances, including enzymes, organic acids, and secondary metabolites, which affect both the relics themselves and their microenvironments [17,18].

In comparison to synthetic drugs, plant-derived agents are characterized by greater safety, reliability, and environmental friendliness [19]. Plant secondary metabolites include volatile oils (such as geraniol, carvacrol, thymol, and cinnamaldehyde), alkaloids, sulfur-containing compounds (such as allicin), terpenoids, and flavonoids, among others. Many scholars have applied plant-derived fungicidal agents to antifungal research in fields such as food, agriculture, and daily life, all achieving favorable results [20,21,22,23,24]. In the field of cultural heritage protection, Huang demonstrated that cinnamaldehyde exhibits excellent antifungal activity against the major pathogenic fungus affecting the Nanhai No.1, a sunken wooden ship [25]. Wang found that allicin achieved favorable results in volatile antifungal experiments targeting fungi isolated from earthen sites [26]. Additionally, Wang conducted antifungal tests using carvacrol on fungi isolated from shadow puppet artifacts, and carvacrol demonstrated antifungal efficacy comparable to chemical fungicides. These fundings highlights the great potential and promising application prospects of plant-derived fungicidal agents for the preservation of cultural heritage and relics.

The Western Han Dynasty M2 Tomb, located near Lingsheng Lake in Dingtao, Shandong Province, ranked among the Top 10 New Archaeological Discoveries of China in 2012 and was included among the Major Historical and Cultural Sites Under State Protection in 2013. To date, it remains the largest, highest-status, and best-preserved excavated Huangchangticou tomb—a burial structure distinguished by walls constructed from cypress heartwood. The tomb had long been submerged in groundwater until its excavation, and the wood suffered vast environmental change during the excavation process. During the initial excavation phase, the dominant fungus on the surface of the tomb was *Hypochnicium* [27]. Subsequently, the wooden components of the Western Han Dynasty Dingtao M2 Tomb underwent disassembly. After the top layer of the wood was removed, the dominant fungus shifted to *Dacrymyces stillatus* in July 2021, followed by a further change to *Talaromyces pinophilus* in August 2022 [28]. This research focused on the disassembled wood stored in the preservation room. In July 2023, microbial deterioration characterized by pale green and white growth colonies was observed on the surface of the disassembled wooden artifacts stored in the preservation room.

In this study, metagenomic analysis was used to characterize the species and functional composition of microbial communities on wood surface samples collected from different areas in the wood preservation room of the Dingtao M2 Tomb in July 2023. Further research was conducted on their acid-producing capacity and metabolic patterns using targeted metabolomic analysis of organic acids. Finally, antimicrobial experiments were carried out using plant-derived antifungal agents. This study proposes a putative mechanism underlying the degradation of wood in the Western Han Dynasty M2 Tomb in Dingtao by the microbial community, holding significant importance for the long-term preservation of wood. Additionally, it provides fundamental data for the application of plant-derived fungicide agents in addressing the microbial deterioration of organic cultural heritages and relics.

## 2. Results

### 2.1. Scanning Electron Microscope (SEM) Observation of Surfaces of Wood in Preservation Room

**Figure 1:**
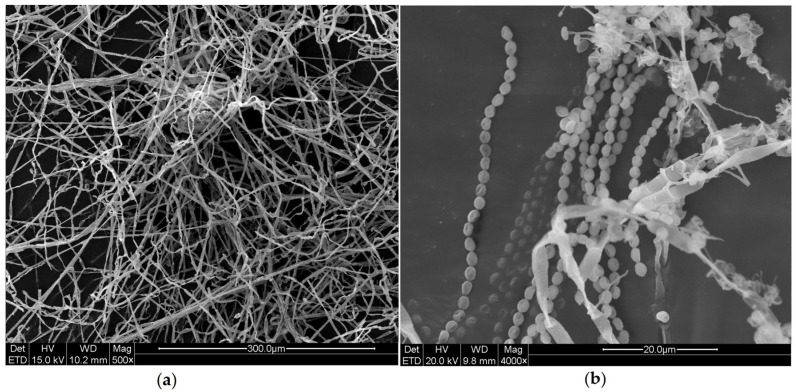
SEM observations of wood surface in the wood preservation room of Dingtao M2 Tomb: (**a**) entangled hyphae; (**b**) spores.

SEM observations revealed abundant spores and hyphae on the wood surfaces and in their crevices (Figure 1). Therefore, we can confirm that the primary contamination is caused by fungi.

### 2.2. Fungal Species Composition Analysis

**Figure 2:**
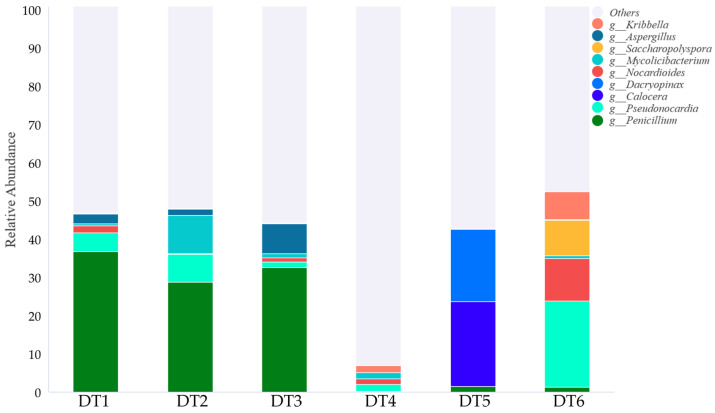
Proportions of the top 9 fungal genera on wood surfaces in the preservation room of Dingtao M2 Tomb (genus level, July 2023).

Species annotation of high-throughput sequencing data from the metagenomic libraries was performed, and a diagram (Figure 2) was generated based on the abundance of the top 9 genera (Table S1). As shown in the diagram, the species composition differed significantly among samples DT1~3, DT4, and DT5~6. *Penicillium* spp. was the most dominant genus in samples DT1~3, accounting for over 28.00%. *Calocera* spp. and *Dacryopinax* spp. were dominant genera in sample DT5, with proportions of 22.00% and 18.78%, respectively. *Pseudonocardia* spp., accounting for 22.25%, was the most dominant genus in sample DT6. Sample DT4 exhibited the highest microbial community diversity and the most uniform species distribution according to the alpha-diversity indices (Table S2); thus, no obvious dominant species were observed in the chart.

### 2.3. Microbial Functional Composition Analysis

**Figure 3:**
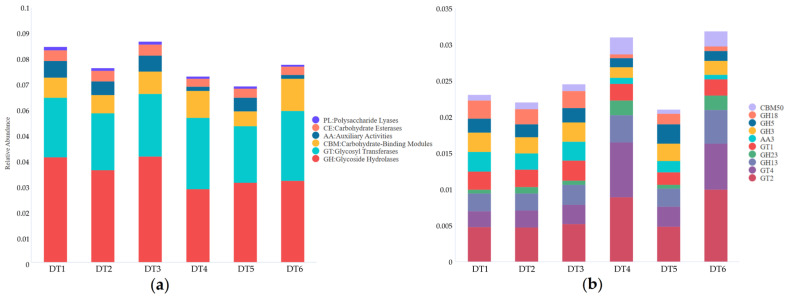
Functional abundance of samples in the CAZy database: (**a**) level 1; (**b**) level 2.

The KEGG database annotations indicate that carbohydrate metabolism is highly active in the six samples, particularly in DT4 and DT6 (Figure S1). The CAZy database annotation analysis further elucidated the detailed characteristics of carbohydrate metabolic activities (Figure 3). At level 1, the six samples show high abundances of enzymes related to glycoside hydrolases (GH) and glycosyl transferases (GT). DT1~3 exhibit more active activities compared to DT4~6. At level 2, it can be more clearly observed that GH5, which is related to cellulose degradation, and AA3, which is associated with lignin degradation, account for a relatively high proportion. Moreover, DT1~3 demonstrate more active cellulose and lignin degradation capabilities than DT4~6. The eggNOG database annotation validated the results from the KEGG and CAZy databases. Additionally, attention should be paid to the high proportion of the “secondary metabolite biosynthesis” function in the level 1 data (Figure S2).

### 2.4. Identification and Morphological Observation of DTP_1

**Figure 4:**
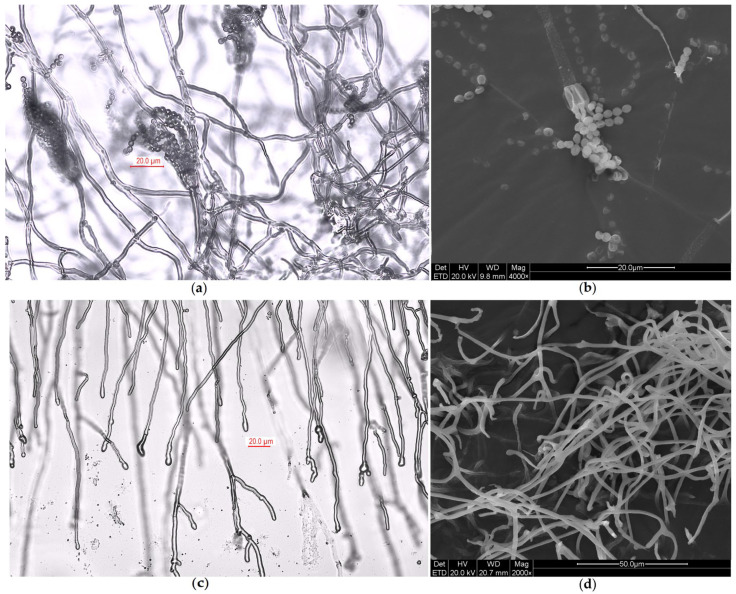
Microscopic morphology of DTP_1. (**a**) Spores and conidiophore under LM; (**b**) spores and conidiophore under SEM; (**c**) hyphae under LM; (**d**) hyphae under SEM.

Based on the high-throughput sequencing results in Section 2.2, we determined that the dominant microorganisms causing damage in the DT1~3 samples were from the genus *Penicillium* spp. We further isolated used light microscopy (LM) and SEM to observe the microscopic morphology (Figure 4).

We further identified the dominant fungal isolate, designated DTP_1, and 20 sequences with 94.00–99.31% similarity to DTP_1 were selected for phylogenetic tree construction. The results with the highest similarity and the phylogenetic tree are shown in Table 1 and Figure S3, respectively. Strain DTP_1 clustered with *Penicillium charlesii* (NG_069647.1) in the phylogenetic tree, supported by a 98% bootstrap value (≥70% indicates high clade reliability). It formed separate, distantly related clades from other reference strains. Based on morphological observation, homology alignment, and phylogenetic analysis, the target strain was taxonomically classified as *Penicillium charlesii*.

### 2.5. SEM Results of Degradation Simulation Experiment

**Figure 5:**
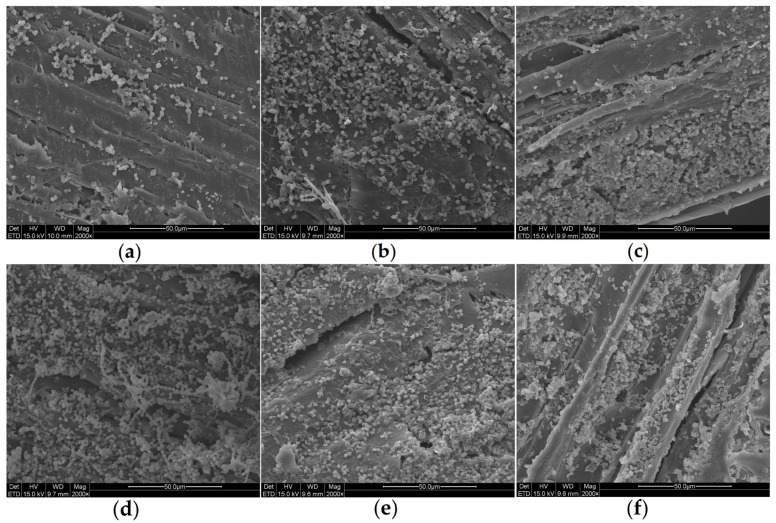
SEM observations on the surfaces of wood samples in DTP_1 degradation simulation experiment (28 °C): (**a**) 30 days; (**b**) 60 days; (**c**) 90 days; (**d**) 120 days; (**e**) 150 days; (**f**) 180 days.

In the experiment, we conducted SEM observations on the surfaces of the wood samples (Figure 5). It was observed that over time, a large number of spores and hyphae gradually appeared on the sample surface. A great quantity of spores also accumulated in the crevices on the wood surface, eventually covering the entire surface of the wood. However, no obvious structural degradation of the wood samples was observed.

### 2.6. Cellulase, Hemicellulase, and Ligninase Activity Detection of DTP_1

We used CMC medium, Xylan medium, and PDA–guaiacol (PDA-G) medium to detect the capabilities of cellulase, hemicellulase, and ligninase. There were no transparent zones around colonies in CMC and Xylan media (Figure S4a,b). Meanwhile, the PDA-G medium showed no color change (Figure S4c). Therefore, we concluded that no cellulase, hemicellulase, or ligninase activity was detected in DTP_1.

### 2.7. Qualitative and Quantitative Detection of Organic Acids Produced by DTP_1

#### 2.7.1. Results of Determination on pH Value Changes in Fermentation Supernatant

**Figure 6:**
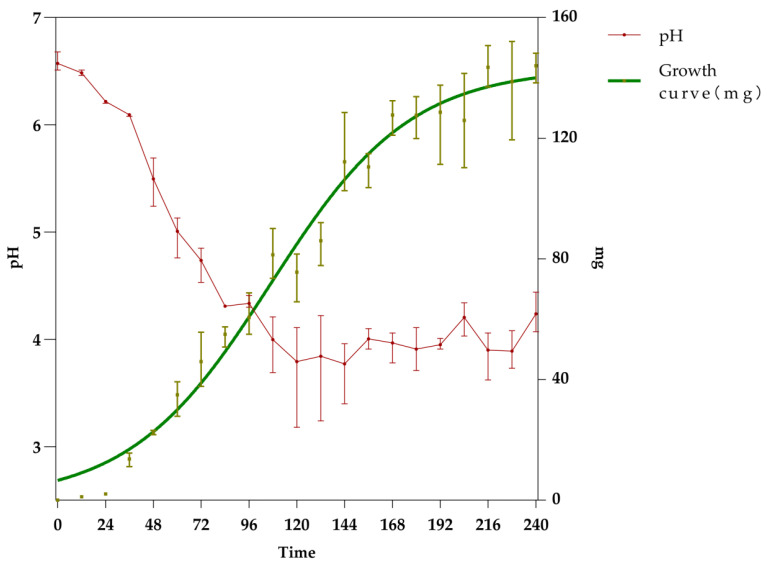
Determination results of pH value (R^2^ = 0.8701) and the growth curve (R^2^ = 0.9657) of the DTP_1 fermentation supernatant.

During the 10-day liquid culture of DTP_1, pH measurements were taken every 12 h with three replicates set up and the nonlinear fitting results were presented with a 95% confidence interval to verify the reliability of the model. The pH value exhibited an S-shaped downward trend corresponding to the ascending phase of the strain’s growth curve (Figure 6). The initial pH value of the culture medium was 6.5, with a maximum decrease of 2.32 (dropping to pH 3.18 at 120 h), and the exponential phase of acid production occurred at 107.1 h (Table S3). The results indicate that the fermentation supernatant contains acidic substances produced by DTP_1, which may be related to the strain’s growth.

#### 2.7.2. Targeted Organic Acid Metabolome Analysis

**Figure 7:**
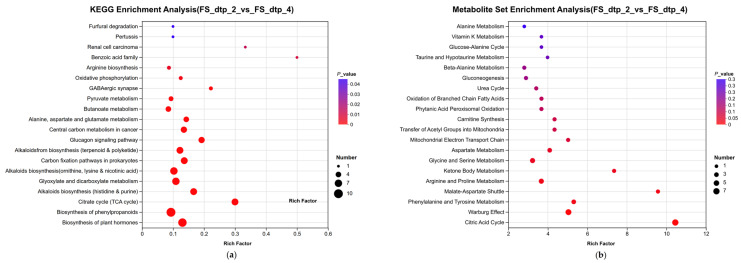
Pathway annotation and enrichment analysis (**a**) KEGG; (**b**) MSEA (a higher Rich Factor means greater metabolite abundance changes and higher pathway activity under current conditions; larger dots indicate more differential metabolites and a more concentrated distribution in the pathway).

Principal Component Analysis (PCA) and sample clustering analysis were performed on the targeted organic acid metabolome across five time points, and the results are presented in Figure S5. The samples exhibited no abnormal distribution under the 95% confidence level, with a high degree of dispersion between groups while showing a certain temporal correlation. These findings confirm the reliability of the selected time points, which can therefore be used as key time points for further analysis. Pathway annotation and enrichment analysis were performed for FS_DTP_2 and FS_DTP_4 (Figure 7), as these two time points were close to the inflection point of the pH variation curve; moreover, significant differences were observed in the clustered heatmap of correlation analysis. Combining the results of both analyses, the most noteworthy finding (at a significance level of *p* < 0.05) is that, between the two time points, the citrate cycle (TCA cycle) and benzoic acid family pathways exhibited the largest magnitude of changes in their associated organic acids. Meanwhile, the biosynthesis of phenylpropanoids and phenylalanine and tyrosine metabolism pathways showed the greatest number of differentially expressed organic acids.

#### 2.7.3. Qualitative and Quantitative Analysis of Targeted Organic Acids

**Figure 8:**
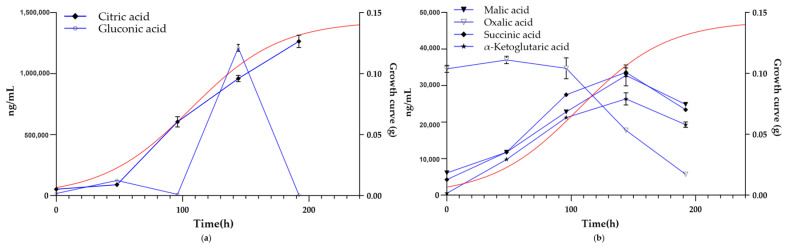
Correlation diagram between the quantitative analysis of 6 key organic acids (blue line) and the growth curve of DTP_1 (red line). (**a**) Citric acid and gluconic acid curves; (**b**) malic acid, oxalic acid, succinic acid and α-ketoglutaric acid.

Six key organic acids with significant fluctuations and correlation with the degradation of wooden cultural relics were screened out, like citric acid, gluconic acid, oxalic acid, malic acid, succinic acid and α-ketoglutaric acid. The content variation curves and growth curve are presented in Figure 8. The variation curves of other non-key organic acids are provided in the supporting material (Figure S6). Relative content analysis (compared with the initial state 0 h) revealed that large amounts of citric acid and gluconic acid were produced during the growth of DTP_1, which we consider to play a pivotal role in the pH decline of the fermentation supernatant and the growth of DTP_1. Both acids began to be rapidly and massively produced after DTP_1 exited the lag phase, while gluconic acid was largely consumed when the growth entered the stationary phase. Oxalic acid was abundant in the early phase of DTP_1 growth and gradually decreased during the exponential growth phase, whereas malic acid, succinic acid and α-ketoglutaric acid exhibited a similar variation trend to gluconic acid but with a much smaller variation range.

### 2.8. Antifungal Efficacy of Fungicidal Agents

#### 2.8.1. Liquid Diffusion Method

**Figure 9:**
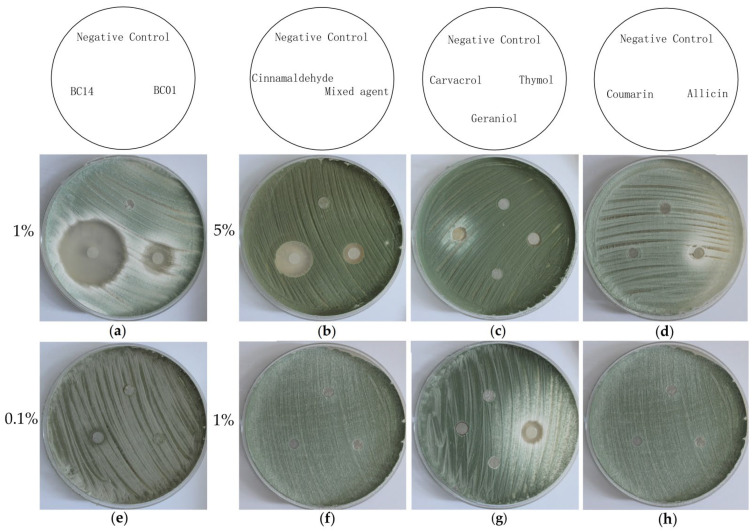
The results of antifungal inhibition assays via the liquid diffusion method. (**a**) 1%BC14 on the left and 1%BC01 on the right; (**b**) 5% cinnamaldehyde the left and 5% mix agent on the right; (**c**) 5% carvacrol on the left and 5% thymol on the right; (**d**) 5% coumarin on the left and 5% allicin on the right; (**e**) 0.1%BC14 on the left and 0.1%BC01 on the right; (**f**) 1%BC14 on the left and 1%BC01 on the right; (**g**) 1% carvacrol on the left and 1% thymol on the right; (**h**) 1% coumarin on the left and 1% allicin on the right.

The results of antifungal inhibition assays via the liquid diffusion method are shown in Figure 9. Chemical antifungal agents exhibited significant inhibitory activity at a concentration of 1%, but their efficacy was drastically reduced when the concentration was lowered to 0.1%. Among the plant-derived fungicide agents, the most effective one is cinnamaldehyde at a 5% concentration, where the largest inhibition zone is observed. At 5% concentration, the mixed agent, carvacrol, and allicin also show certain antifungal activity; however, they basically lose their efficacy when the concentration is reduced to 1%. Interestingly, thymol displays poor activity at 5% concentration but exhibits better antibacterial effects after concentration reduction. Coumarin shows no antibacterial activity at all.

#### 2.8.2. Gas Volatilization Method

**Figure 10:**
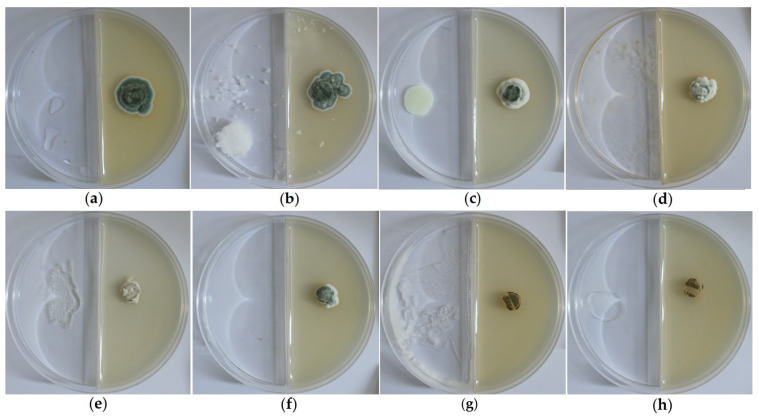
The results of gas volatilization fungicide experiment. (**a**) Negative control; (**b**) coumarin; (**c**) cinnamaldehyde; (**d**) carvacrol; (**e**) allicin; (**f**) geraniol; (**g**) thymol; (**h**) mixed agent.

The results of antifungal inhibition assays via the gas volatilization method are shown in Figure 10. Under the condition that negative controls performed normally, the volatile gases of the mixed agent and thymol exhibited significant antifungal activity. The volatile gases of allicin and geraniol also showed excellent antifungal effects, while those of cinnamaldehyde and carvacrol, though effective, were slightly weaker than the aforementioned agents. In contrast, the inhibitory effect of coumarin was not obvious.

## 3. Discussion

The Western Han Dynasty Dingtao M2 Tomb is experiencing microbial degradation characterized by pale green and white growths on the surface of the wood stored in its preservation room, observed in July 2023. In this study, metagenomic analysis was used to characterize the species and functional composition of the microbial community, and in further research, a targeted metabolomic analysis of the organic acids was conducted to investigate their acid-producing capacity and metabolic patterns. In the bar chart of species abundance at the genus level, we found that the species composition differed significantly among samples DT1~3, DT4, and DT5~6. In samples DT1~3, *Penicillium* spp. was the most dominant genus, accounting for more than 28.00% of the community. In sample DT5, *Calocera* spp. and *Dacryopinax* spp. were the dominant genera, with relative abundances of 22.00% and 18.78%, respectively. In sample DT6, *Pseudonocardia* spp. was the most dominant genus, representing 22.25% of the community.

**Figure 11:**
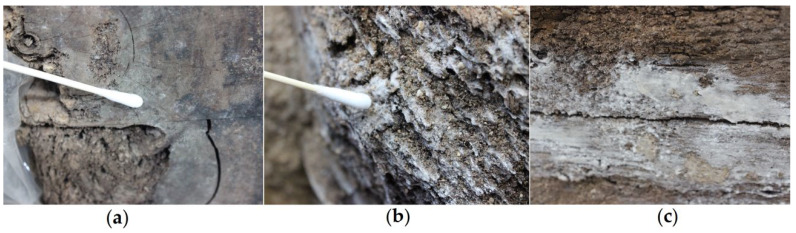
Microbial degradation on wood surfaces in the preservation room of the Dingtao M2 Tomb: (**a**) pale green microbial contamination; (**b**) sparse white plaque; (**c**) dense white plaques.

The wood was dried using different methods: wrapping with plastic wrap, burial in sand, and stacking and covering with plastic sheeting. Therefore, differences in these preservation procedures led to variations in the main microorganisms among these three areas. Wang was the first to identify *Dacrymyces* sp. in a study on tomb chamber wood; hence, it is understandable that *Dacrymyces* sp. served as the main pathogenic microorganism in the samples from the stacked wood. Furthermore, the other dominant fungal genus identified, *Calocera* spp., belongs to the same family (Dacrymycetaceae) as *Dacrymyces* spp. In addition, *Calocera* spp., *Dacrymyces* spp., and *Pseudonocardia* spp. exhibit strong wood-degrading capabilities. A 2022 species abundance study detected *Penicillium* spp. at a relatively low abundance [28]. Compared with the storage conditions in 2017 (10~16 °C, waterlogged) [27] and 2022 (27.5 °C, 70%) [28], the DT1~3 samples were collected from wooden surfaces exposed to different temperatures and humidities. Consequently, our research focused on *P. charlesii* DTP_1, which we isolated from the end of the wood placed in the shelf area and cultured under laboratory conditions. In the on-site inspection images (Figure 11), pale green mycelial plaques can be clearly observed on the end of the wood. Under SEM observations of both the collected wood samples and the wood samples in simulation experiments (Figure 5), we identified the spores and hyphae of DTP_1, with some accumulating and growing in the gaps of the wood—these all pose potential risks. *P. charlesii* has been isolated from soil and/or sediment samples in three caves in the Tatra Mountains, Slovakia [29]. This suggests that the *P. charlesii* present on the wood surface may originate from the burial soil or groundwater. Additionally, the environment in the preservation room is conducive to its growth, allowing it to proliferate visibly. Most *Penicillium* species primarily reproduce asexually, and aerial conidia can germinate again into mycelia under suitable environmental conditions.

As a brief summary, there may be three factors that contributed to the dominance of *Penicillium* on the surface of the end of the wrapped wood: (1) changes in environmental temperature and humidity; (2) the well-ventilated environments facilitating the dispersal of spores; and (3) the strong dispersal ability of *Penicillium*. Therefore, immediate measures should be taken to control the environmental conditions in the preservation room to prevent further spread of *Penicillium* contamination.

Microorganisms typically produce enzymes to break down and utilize the organic components from wood or wooden artifacts. However, our preliminary experiment confirmed that DTP_1 does not possess this capability (Figure S4). Therefore, we focused our research on the degradation mechanisms of other metabolites: organic acids. Shaking cultivation in PDA medium provided an optimal environment for the natural growth of DTP_1. Under this condition, the organic acid production of DTP_1 suggested its inherent growth trait. The acid-producing capacity was characterized by pH variation in the culture supernatant, which revealed a strong correlation between acid production and microbial growth—acid production reached its peak during the most vigorous growth phase.

The organic acids produced by *Penicillium* spp. typically include oxalic acid, citric acid, succinic acid, and gluconic acid, among others [30,31,32]. Qualitative and quantitative analysis of organic acids indicated that the process of major organic acid production by DTP_1 can be summarized as follows: a large amount of oxalic acid is produced during the growth lag phase and gradually decreases at the beginning of the exponential phase. Citric acid, succinic acid, and malic acid gradually accumulate at the early stage of the exponential phase, which may be attributed to microbial growth and the active tricarboxylic acid (TCA) cycle during this period. Gluconic acid begins to accumulate rapidly in the middle of the exponential phase and decreases sharply when growth reaches the stationary phase. Our results revealed that the rapid decrease in pH is mainly related to citric acid and gluconic acid.

These findings indicated that DTP_1 may assist the microbial community in degrading archaeological wood by producing organic acids. We speculate that this may involve two pathways: direct degradation and indirect degradation. (1) Direct degradation: These organic acids create conditions for the acid hydrolysis of wood, rendering wooden cultural heritage and relics more prone to degradation [33]. This is relatively common in brown-rot fungi: organic acids such as humic acid and oxalic acid promote the Fenton reaction by chelating iron, manganese, and copper [34]. For instance, oxalic acid is the most prevalent organic acid secreted by most fungi [35] and participates in nearly all stages of wood decomposition, such as chelating unstable Mn^3+^ ions [36], supplying H_2_O_2_, and lowering the pH of the extracellular environment surrounding the hyphae [37]. Increased wood acidity led to the destruction of carbohydrate-rich layers in wood cells, thereby exacerbating the degradation of cell wall ultrastructure [38]. (2) Indirect degradation: In our simulated experiments, strain DTP_1 alone exhibited extremely limited wood degradation ability, and no obvious degradation was observed under SEM. This may be attributed to the organic acid produced by DTP_1 contributing to indirect degradation rather than direct decay. We hypothesize that on the surface of archaeological wood, which is rich in lignin, the production of organic acids may serve as a preliminary environmental regulatory basis for enzyme production. This provides more readily available products and a favorable environment for enzyme activity for other enzyme-producing microorganisms colonizing the wood surface, such as *Dacrymyces*.

In the differential enrichment analysis of organic acid metabolic sets, utilizing targeted metabolomics (Figure 7), we observed the enrichment of lignin degradation-related metabolic pathways such as benzoic acid during the late growth stage (stationary phase). As far as we are aware, these pathways represent key regulatory modules that are highly associated with organic acid-driven degradation of archaeological wood [39]. In the functional annotations of the metagenome, we found that the microbial community at the end of the wrapped wood was abundant in glycoside hydrolase functions. Based on this, we hypothesize a potential mechanism by which DTP_1 promotes archaeological wood degradation through organic acid production. This involves the interaction of growth and metabolism among different species in the microbial consortia [40]. DTP_1 produces organic acid to lower the pH value on the wood surface, thereby providing a favorable acidic reaction environment [41] for extracellular enzymes (e.g., cellulases, laccases, peroxidases, etc.) produced by functional microorganisms within the community. This leads to the initial degradation of archaeological wood, generating small molecular benzoic acid derivatives such as ferulic acid and vanillic acid [42,43]. DTP_1 then utilizes these small molecules, via its own benzoate pathway and a series of other metabolic pathways, and the products enter the TCA cycle [44]. It may further produce acids such as citric acid to maintain a suitable environment for the surrounding microbial community. The specific mechanism still requires further detailed investigation.

Although chemical agents demonstrated good antifungal ability in the experiment, they cannot be applied in high concentrations due to potential environmental hazards and the principle of minimal intervention in cultural heritage conservation. Consequently, they fail to achieve satisfactory antifungal efficacy when the concentration is reduced, making them less preferable for application. Plant-derived agents are environmentally friendly, and their antifungal effects vary with different application methods. Specifically, cinnamaldehyde is more suitable for liquid-form antifungal applications, whereas allicin and carvacrol are better suited for volatile-gas antifungal applications. Additionally, Corbu found that allicin extract inhibited the adherence capacity and the production of biodeteriogenic products, such as cellulase, organic acids, and esterase [45]. Compared with chemical agents, plant-derived agents exhibit environmental friendliness, relatively favorable effects at low concentrations, and versatility in application methods. However, these agents also have certain limitations. First, some plant-derived agents have aromatic odors, while others can be pungent, such as allicin (volatile sulfur compounds) [46]. Additionally, they are flammable as organic substances. Therefore, when actually applying them to conservation sites, the method of use and safety should be ensured, and further modification of the agents should be carried out.

## 4. Materials and Methods

### 4.1. Sample Collection and Microbial Degradation Investigation

This study conducted microbial degradation investigations in the wood preservation room of the Dingtao M2 Tomb in July 2023, revealing 3 kinds of microbial contamination on the surface of the tomb wood (Figure 11). The environmental temperature in the preservation room was 25 °C with a relative humidity of 87%. The disassembled archaeological wood from the tomb chamber was rinsed and soaked with double-distilled water, then dried under three different conditions according to the condition of the wood samples: (a) wrapped wood: samples were fully wrapped in plastic wrap and placed in the shelf area for natural drying; (b) sand-buried wood: samples were preserved by sand burial drying; (c) stacked wood: samples with a large size were stacked and covered with plastic sheeting and left to dry naturally in the open air in the cleaning area.

Three sites of pale green microbial contamination (DT1~3) were selected from the wrapped wood; one site of sparse white plaque (DT4) was selected from the sand-buried wood; and two sites of dense white plaques (DT5~6) were selected from the stacked wood. All samples were transported to the laboratory in ice packs. Three types of samples were collected from each site as follows:

- Carbon conductive adhesive was used to collect microbial contamination from the wood surface, which was then placed into a sterile EP tube for SEM observation (Section 4.2).
- A cotton swab was used to dip the plaque and streak it on PDA medium for fungal culture and morphological observation (Section 4.3).
- Plaque samples were gently scraped with a sterile scalpel and placed in a sterile EP tube prepared for microbiome analysis (Section 4.4).

### 4.2. SEM Observation

Samples were dried in a dish until completely dry. They were then fixed onto an SEM-specific sample stage, with excess material trimmed to maintain a size of 1 cm × 1 cm. The surface of the prepared samples was subsequently sputter-coated with gold at a current of 24 mA for 300 s. Observations and image recording were performed using an SEM (FEI Quanta 200, Hillsboro, Oregon, USA). The measurement conditions were as follows: EHT: 15.0 kV, WD: 9.6–10.2 mm, and Mag: 0.3 KX–5 K.

### 4.3. Isolation, Purification and Molecular Identification of Fungi

Multiple rounds of traditional streaking culture were used to purify the fungi. Sequence alignment of the ITS1-5.8S-ITS2 region failed to yield clear results. Consequently, the nuclear large subunit (nucLSU) rDNA region amplified via PCR using LROR (5′-ACCCGCTGAACTTAAGC-3′)/LR7 (5′-TACTACCACCAAGATCT-3′) primers was employed for subsequent sequence alignment and phylogenetic analysis. The unpurified PCR products were sent to GENEWIZ (Beijing, China) for sequencing. Then, the sequence homology of the amplified fragments was analyzed using NCBI BLAST (version 2.17.0, https://blast.ncbi.nlm.nih.gov/Blast.cgi?PROGRAM=blastn&PAGE_TYPE=BlastSearch&LINK_LOC=blasthome, accessed on 11 August 2025). A total of 20 sequences sharing 94–99.31% similarity with the target strain were retrieved. Phylogenetic analysis was conducted using MEGA software (version 12) to build a phylogenetic tree via the Maximum Likelihood (ML) method, and the bootstrap test was performed with 1000 replications to validate the topological stability.

### 4.4. Total DNA Extractions and High-Throughput Sequencing

We extracted total genome DNA from 6 samples (DT1~6) using the DNeasy PowerSoil Kit (QIAGEN, Hilden, Germany). The extracted total DNA was then sent to Novogene Genome Sequencing Company (Beijing, China) for quality inspection and metagenome library construction. The constructed libraries were sequenced on the Illumina PE150 platform (San Diego, CA, USA). Subsequent data processing included a series of analyses, such as data filtering, species annotation (based on the Micro_NR database), common functional annotations (including KEGG, eggNOG, and CAZy), and clustering analysis on the Novogene Cloud Platform (https://magic.novogene.com/customer/main#, accessed on 7 August 2025).

### 4.5. Fungal Degradation Simulation Experiment

Residual wood (Artifact No. DDCMLS:1, Populus wood) collected from around the thief hole of the Dingtao M2 Tomb was used for the simulation experiment. The wood samples were cut into pieces measuring 1 cm × 1 cm × 0.3 cm, placed in a glass Petri dish, sterilized twice in an autoclave at 121 °C for 30 min each time, and then dried in an oven. A 100 μL spore suspension (1 × 10^7^ Spores/mL) of DTP_1 was spread on PDA medium and cultured at 28 °C for 3 days. Under sterile conditions, the wood samples were placed at the center of the medium surface using sterile tweezers, followed by incubation at 28 °C for 30, 60, 90, 120, 150, and 180 days. At each time point, the surface of wood samples was observed under SEM according to the method described in Section 4.2.

### 4.6. Cellulose, Hemicellulose, and Lignin Degradation Capability Detection

We used specialized culture media to evaluate the ability of DTP_1 to degrade cellulose and lignin, following the methods described below:

- CMC medium (composition: 0.2% NaNO_3_, 0.05% KCl, 0.1% K_2_HPO_4_, 0.05% MgSO_4_, 0.2% CMC, 0.2% peptone, 2% agar) was used to assess cellulose-degrading capability.
- Xylan medium (composition: 0.5% Xylan, 0.2% NH_4_Cl, 0.1% KH_2_PO_4_, 0.05% MgSO_4_, 2% agar) was used to assess hemicellulose-degrading capability.
- DTP_1 cultures grown at 28 °C for 7 days were spot-inoculated onto the above two mediums. Subsequently, 5 mL of iodine–potassium iodide solution was added to the plate, and then incubated in the dark at room temperature for 5 min. Fungi with cellulose-degrading ability produce transparent zones around their colonies.
- PDA–guaiacol medium (composition: 1.2% potato extract, 2% glucose, 0.04% guaiacol, 2% agar) was used to assess lignin-degrading capability. After spot-inoculating DTP_1 onto the medium, the cultures were incubated at 28 °C for 7 days. Fungi capable of utilizing lignin exhibit a brown discoloration reaction.

### 4.7. Liquid Shaking Culture of DTP_1

We used sterile ddH2O to collect the spores of DTP_1 on culture plats and filtrated through three layers of sterile gauze. Subsequently, spore counting was conducted under a microscope (Nikon E200, Tokyo, Japan) using a haemocytometer, adjusting the suspension to a final concentration of 5 × 10^6^ spores/mL. The spore suspension was inoculated at a 0.5% inoculum rate into 20 mL of sterilized PD medium. Cultures were incubated at 28 °C with shaking at 120 r/min for 10 days, with measurements taken every 12 h, resulting in 20 detection time points total. Three biological replicates were set for each time point.

### 4.8. pH Determination of DTP_1 Fermentation Supernatant

At each sampling time point, 5 mL of DTP_1 fermentation broth was collected and centrifuged at 10,000 rpm for 10 min, and the supernatant was obtained as the test solution. The pH value was measured using a pH meter (Yoke P901, Shanghai, China): the instrument was preheated and calibrated in advance. After the pH electrode was cleaned and dried, it was immersed in the fermentation supernatant, gently swirled, and then allowed to stand. The pH value of the fermentation supernatant was recorded when the reading stabilized.

### 4.9. Qualitative and Quantitative Targeted Metabolomics of Organic Acids

Targeted metabolomics was employed for the qualitative and quantitative analysis of organic acids in fungal fermentation supernatants. The determination was performed by Majorbio Bio-Pharm Technology Co., Ltd. (Shanghai, China). Analysis was conducted via Graphpad Prism (version 10.1.2) and Majorbio Cloud Platform (https://cloud.majorbio.com/, accessed on 16 January 2026). Analyses were performed on a liquid chromatography–electrospray ionization–tandem mass spectrometry (LC-ESI-MS/MS, UHPLC-Qtrap) system (SCIEX QTRAP 6500+) (LC-ESI-MS/MS (UHPLC-Qtrap)). Information on the organic acid standards is provided in Table S4. The total ion current (TIC) chromatograms of standards and samples are shown in Figure S7, which demonstrate excellent resolution and good peak shapes for all organic acids. Standard linear regression calibration curves were constructed by plotting the ratio of the chromatographic peak area of target standards to that of the isotope-labeled internal standards (ISs, including succinic acid-D4, cholic acid-D4, and salicylic acid-D4) as the ordinate against concentration as the abscissa. All target organic standards showed excellent linearity with a correlation coefficient (R^2^) > 0.99. For quantification, the peak area ratio of target analytes to IS in samples was substituted into the calibration curve equations to calculate the target concentrations, which were further converted to actual contents based on the sample volume. Key organic acids with significant content fluctuations and relevance to wooden cultural relic degradation were screened.

- Sample and standard preparation: Based on the variations in pH and growth curve of DTP_1, 5 time points (0 h, 48 h, 96 h, 144 h, and 192 h) were selected. Three replicate samples were set for each time point. Fungal fermentation broth samples were centrifuged at 12,000× *g* for 15 min at 4 °C to remove mycelia and insoluble impurities, and the supernatant was filtered through a 0.22 μm organic phase syringe filter to obtain the 15 analytical samples through the following method: Accurately pipette 50 μL fungal fermentation broth samples into a centrifuge tube, then add 20 μL of the IS working solution, 50 μL methanol–water and 200 μL of methanol/acetonitrile mixture (1:1, *v*/*v*). Vortex the mixture for 1 min. Subsequently vortex the mixture for 1 min, then centrifuge at 14,000 rcf and 4 °C for 20 min. Pipette 160 μL of the supernatant into another tube. Accurately weigh 67 organic acid standards and the IS. Dilute with 50% methanol–water separately to appropriate concentrations to create working standard solutions. Pipette 50 μL of the organic acid working standard solutions into a centrifuge tube, then add 10 μL of the IS working solution and 100 μL of methanol/acetonitrile mixture (1:1, *v*/*v*). Vortex the mixture for 1 min. All samples had 20 μL of 200 mM 3-nitrophenylhydrazine hydrochloride (3NPH·HCl) and 20 μL of 120 mM 1-ethyl-3-(3-dimethylaminopropyl) carbodiimide hydrochloride (EDC·HCl, containing 6% pyridine) added. Vortex the reaction system for 30 s, perform a brief centrifugation for 5 s to collect the solution, and incubate the mixture at 40 °C for 60 min. Subsequently vortex the mixture for 30 s, then centrifuge at 14,000 rcf and 4 °C for 20 min. Finally, transfer the supernatant carefully to a vial for instrumental analysis.
- LC-ESI-MS/MS (UHPLC-Qtrap) system: Chromatographic conditions: The separation was conducted using an ExionLC AD system equipped with a Waters HSS T3 column (2.1 × 150 mm, 1.8 μm) (Milford, MA, USA). The column temperature was maintained at 40 °C, and the injection volume was set at 2 μL. The mobile phase consisted of phase A (0.03% formic acid aqueous solution) and phase B (0.03% formic acid methanol solution), using the following gradient elution program: 0.0–1.5 min, 98% A; 1.5–4.0 min, 80% A; 3.0–4.0 min, 80% A; 4.0–4.5 min, 77% A; 4.5–6.5 min, 65% A; 6.5–12 min, 60% A; 12–13 min, 0% A; 13–15 min, 98% A, with a flow rate of 0.45 mL/min. Mass spectrometry was carried out on the SCIEX QTRAP 6500+ (Shanghai, China) operating in the negative ion mode. The optimized MS parameters were set as follows: curtain gas (CUR) = 35, collision-activated dissociation (CAD) = medium, ion spray voltage = −4500 V, source temperature (TEM) = 500 °C, ion source gas 1 (GS1) = 55, and ion source gas 2 (GS2) = 55.

### 4.10. Targeted Metabolome Analysis

For the raw targeted metabolomic data of organic acids, missing value imputation and subsequent data transformation were conducted to ensure the reliability of subsequent statistical analysis [47,48,49]. The hypergeometric distribution algorithm was applied to identify the KEGG pathways with significantly enriched metabolites in the metabolome, and the Benjamini–Hochberg (BH) method was used for *p*-value correction. Additionally, the Small Molecule Pathway Database (SMPDB) was employed to conduct Metabolite Set Enrichment Analysis (MSEA) on all metabolites identified in the two groups, so as to determine and interpret the variation patterns of metabolite concentrations in key biological pathways and obtain information on the pathways with significantly enriched metabolites.

### 4.11. Fungicidal Activity Assay of Plant-Sourced Fungicides

We evaluated the fungicidal activity of 2 chemical agents, 6 plant-derived agents, and 1 mix agent (Table 2) using two simulated application methods: liquid diffusion and gas volatilization.

- Liquid diffusion: We use ddH_2_O and DMSO solvent (1% dimethyl sulfoxide (DMSO), 0.1% Tween-80, and ddH_2_O) to dilute agents. A total of 100 μL of spore suspension (5 × 10^6^ spores/mL) prepared as described in Section 4.7 was added to PDA medium and spread evenly. Sterilized triple-layer filter paper discs (d = 4 mm) were placed on the medium surface, and the fungicide solutions or negative control solutions were dropped onto the discs. Cultures were incubated at 28 °C for 5 days. The fungicidal activity of each agent was indicated by the diameter of the inhibition zone.
- Gas volatilization: The volatile fungicidal activity was determined using split-plot plates. PDA medium was poured into one compartment; a fungal lawn approximately 8 mm in size was placed upright on one side of the culture medium. For each plate, 50 μL of the fungicide stock solution was added to the other compartment, resulting in a fungicide air concentration of 625 μL/L (based on the total volume of approximately 80 mL in a 90 mm Petri dish). An equal volume of sterile distilled water was used as the negative control to assess the effect.

## 5. Conclusions

Some of the preserved tomb wood from the Dingtao Han Tomb was at risk of acidification and accelerated degradation by microorganisms. The dominant fungus *Penicillium charlesii* DTP_1 isolated from the end of the wrapped wood, which assists the microbial consortium in degrading archaeological wood by producing organic acids, indicates a mechanism in which microbial communities synergistically degrade. The primary degradation challenges faced by the wood vary depending on storage environments and the duration elapsed since various conservation treatments, highlighting the necessity of real-time monitoring and preventive conservation measures. Compared with chemical agents, plant-derived agents exhibit better application prospects. However, further optimization of the plant-derived agents and application methods are still required for their practical implementation. This study provides a scientific basis for the preservation of wood from the Dingtao M2 Han Tomb, establishes a theoretical foundation for the conservation of wooden cultural heritage and artifacts, and offers new insights and directions for optimizing artifact preservation environments and developing antifungal agents.

## Figures and Tables

**Table 1:** Molecular identification of strains isolated from DT1~3.

Fungus	Closest Relative Strain	Phylum	Similarity	Accession Number
DTP_1	*Penicillium charlesii*	Ascomycota	99.31%	NG_069647.1

**Table 2:** Fungicides employed in this research.

	Agent	Main Isothiazolinone	Solvent	Concentration
Chemical agents	BC01	3% Isothiazolinone	ddH_2_O	0.1%, 1%
	BC14	14% Isothiazolinone	ddH_2_O	0.1%, 1%
	Coumarin	2H-Chromen-2-one	DMSO	1%, 5%
	Cinnamaldehyde	3-Phenyl-2-propenaldehyde	DMSO	1%, 5%
	Allicin	Diallyl thiosulfinate	DMSO	1%, 5%
Plant-derived	Thymol	2-Isopropyl-5-methylphenol	DMSO	1%, 5%
	Carvacrol	2,6-Dimethyl-5-isopropylphenol	DMSO	1%, 5%
	Geraniol	3,7-Dimethyl-2,6-octadien-1-ol	DMSO	1%, 5%
	Mix agent	Geraniol: Carvacrol: Thymol = 4:4:2	DMSO	1%, 5%

## Data Availability

The raw data are available in the China National Center for Bioinformation database under Bioproject accession number PRJCA059618. (https://ngdc.cncb.ac.cn/search/all?q=PRJCA059618, accessed on 9 March 2026).

## Supplementary Materials

The following supporting information can be downloaded at https://www.mdpi.com/article/10.3390/ijms27073233/s1.
