# Supplementary material for: Preliminary Study on the Synergistic Degradation Mechanism of the Microbial Community on the Wood of the Dingtao M2 Tomb
Source: Int J Mol Sci. 2026 Apr 2;27(7):3233. doi: 10.3390/ijms27073233 (PMC13073937; doi:10.3390/ijms27073233)
Supplement: Supplementary file 1 [file ijms-27-03233-s001.zip › ijms-4230429-supplementary.pdf]

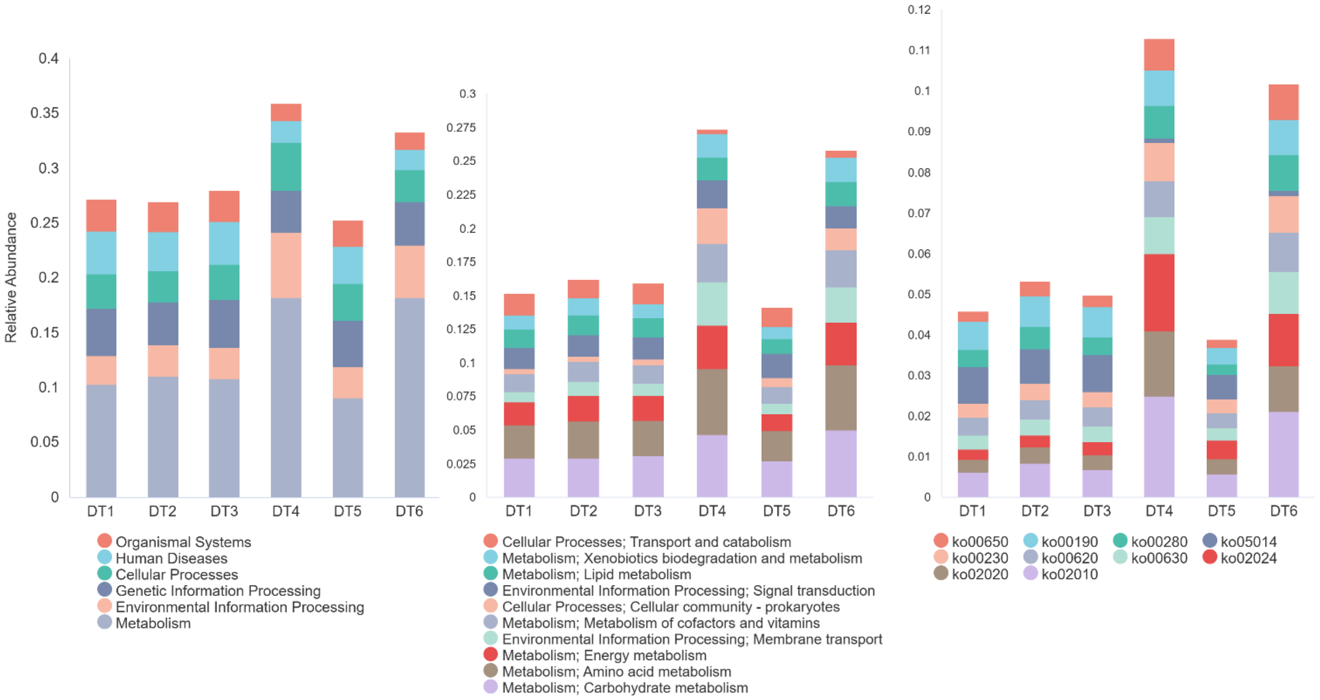

(a) (b) (c)

Figure S1. Functional abundance of wood surface samples in the KEGG database：(**a**) level 1; (**b**) level 2; (**c**) level 3.

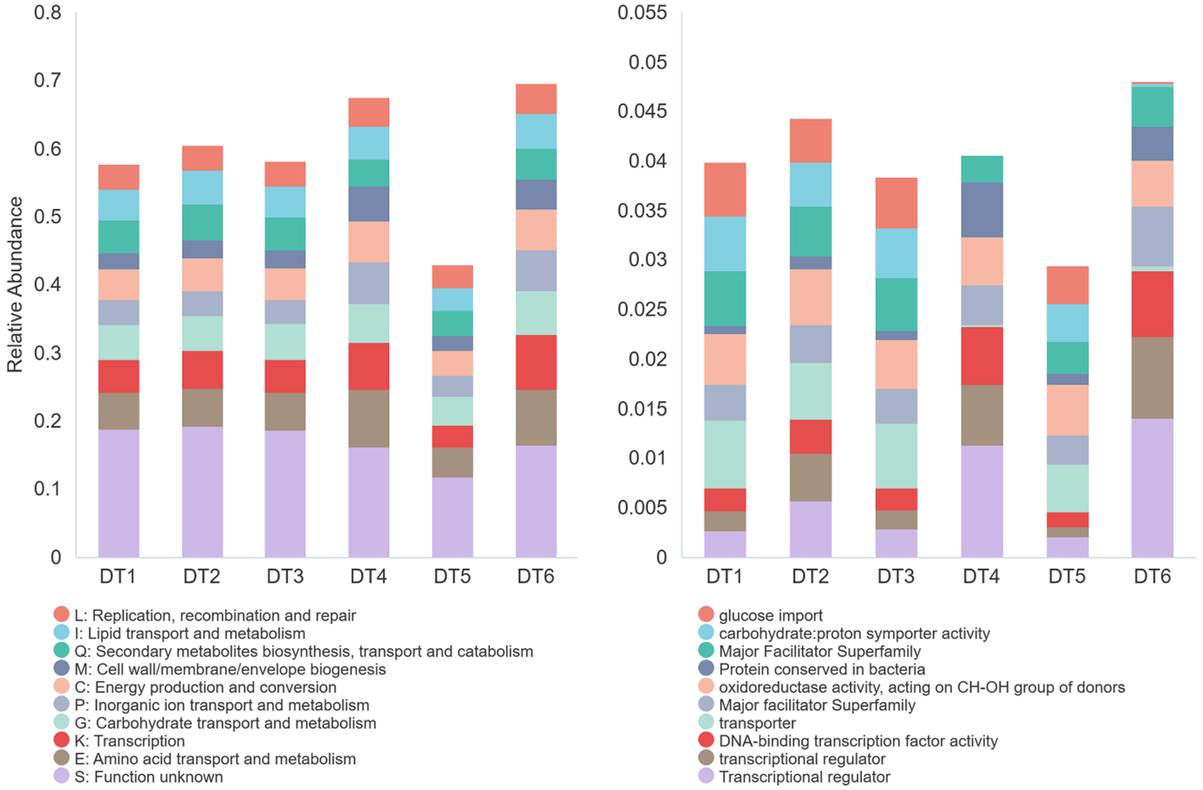

(a) (b)

Figure S2. Functional abundance of wood surface samples in the egg database：(**a**) level 1; (**b**) level 2.

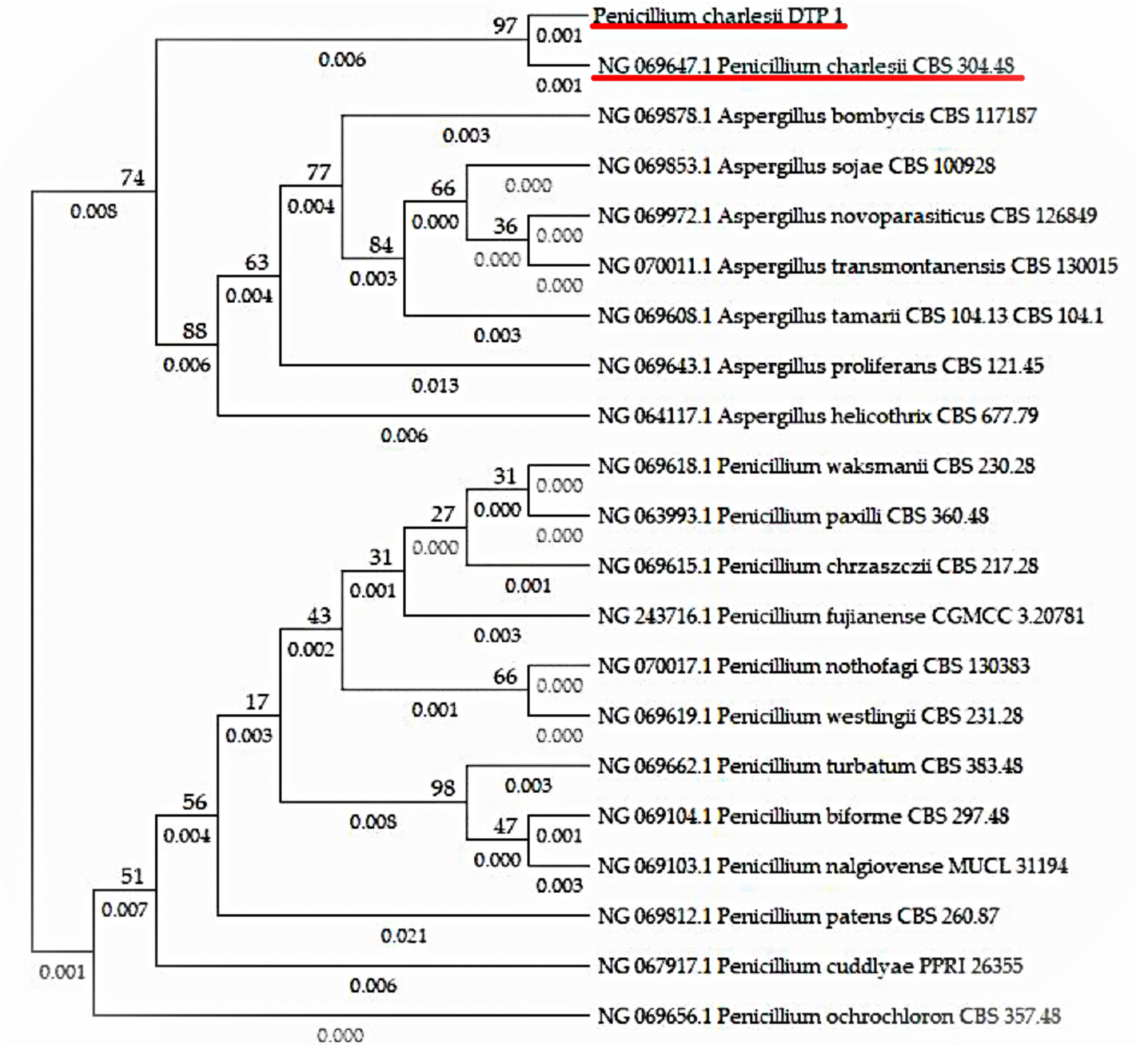

Figure S3. The phylogenetic tree of DTP-1.
(A total of 20 sequences sharing 94%–99.31% similarity with the target strain were retrieved to conduct the phylogenetic analysis, using MEGA software via the Maximum Likelihood (ML) method, and the bootstrap test was performed with 1000 replications.)

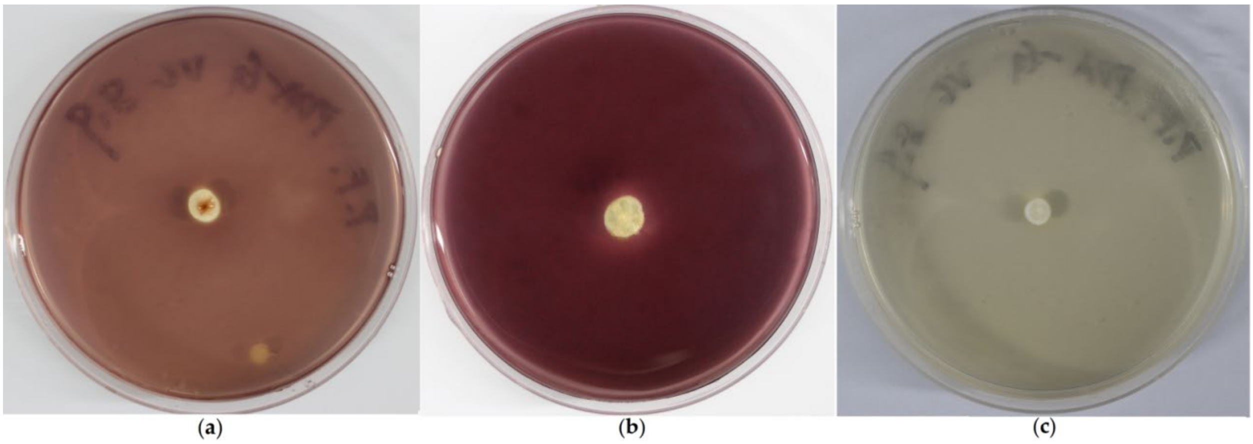

Figure S4. The result of detection of cellulase, hemicellulase, and ligninase activity of DTP-1. (**a**) colony in CMC medium after reaction; (**b**) colony in Xylan medium after reaction; (**c**) colony in PDA-G medium.

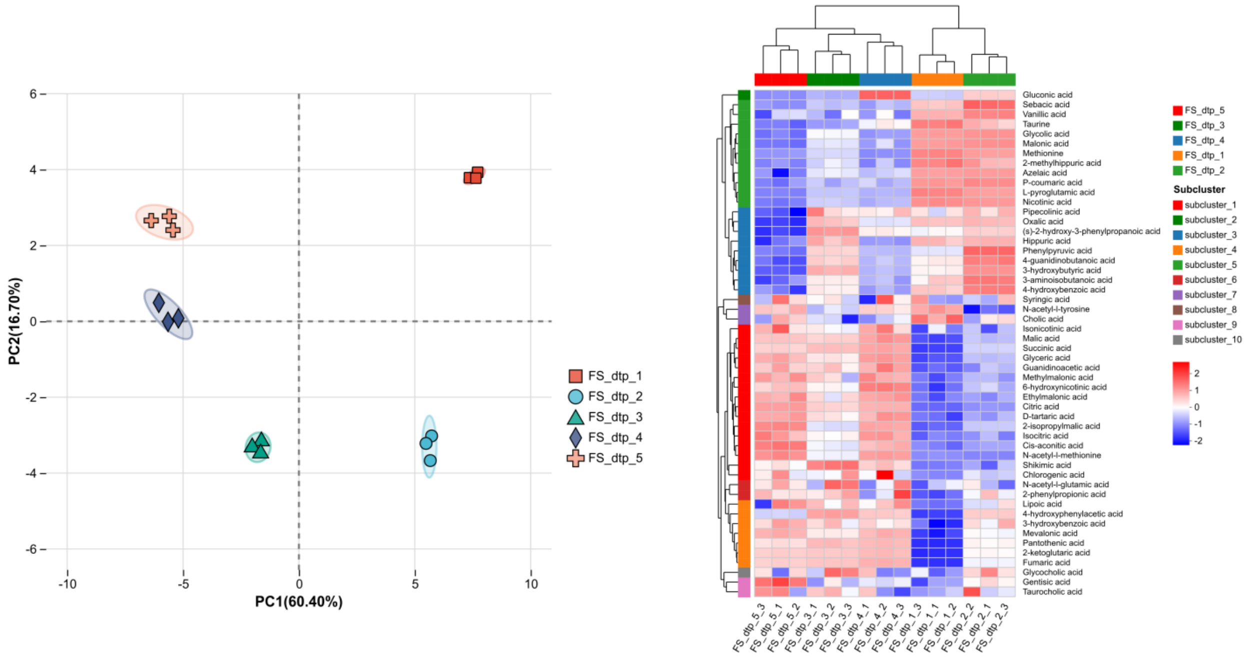

(**a**) (**b**)

Figure S5. Principal Component Analysis (PCA) and sample clustering analysis between FS_DTP_2 and FS_DTP_4: (**a**) PCA; (**b**) sample clustering analysis.

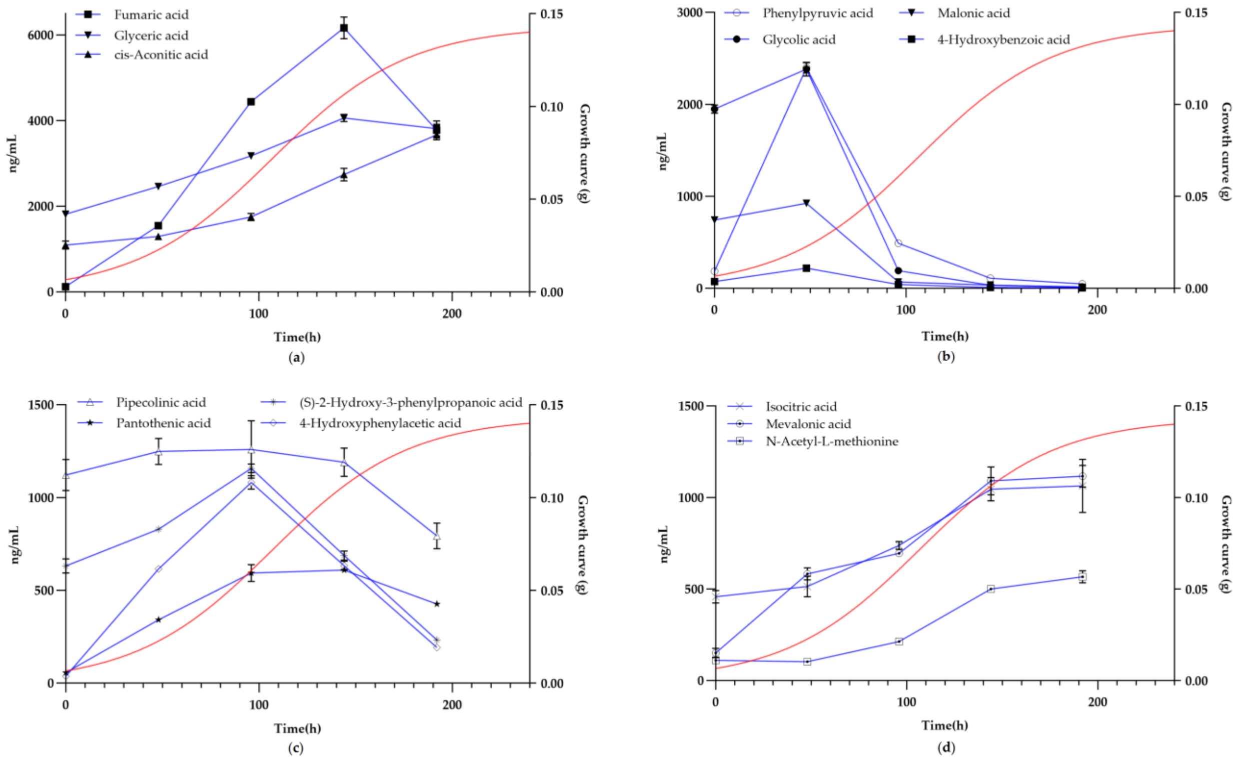

Figure S6. The variation curves of organic acids which are not shown in the text.

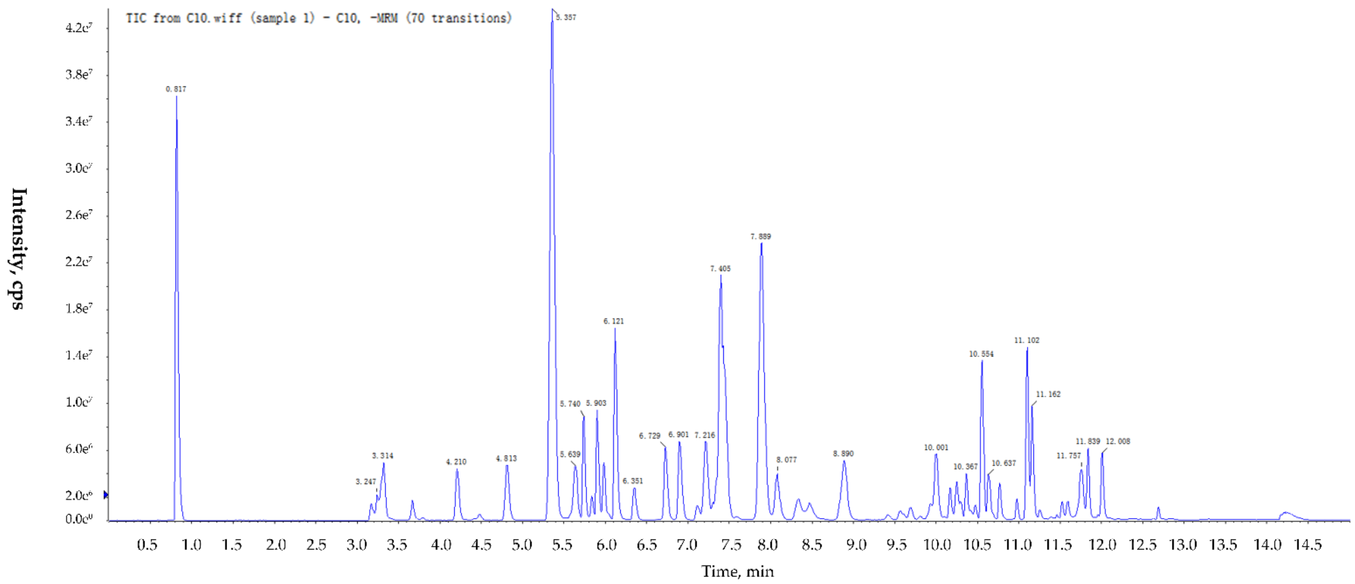

(**a**)

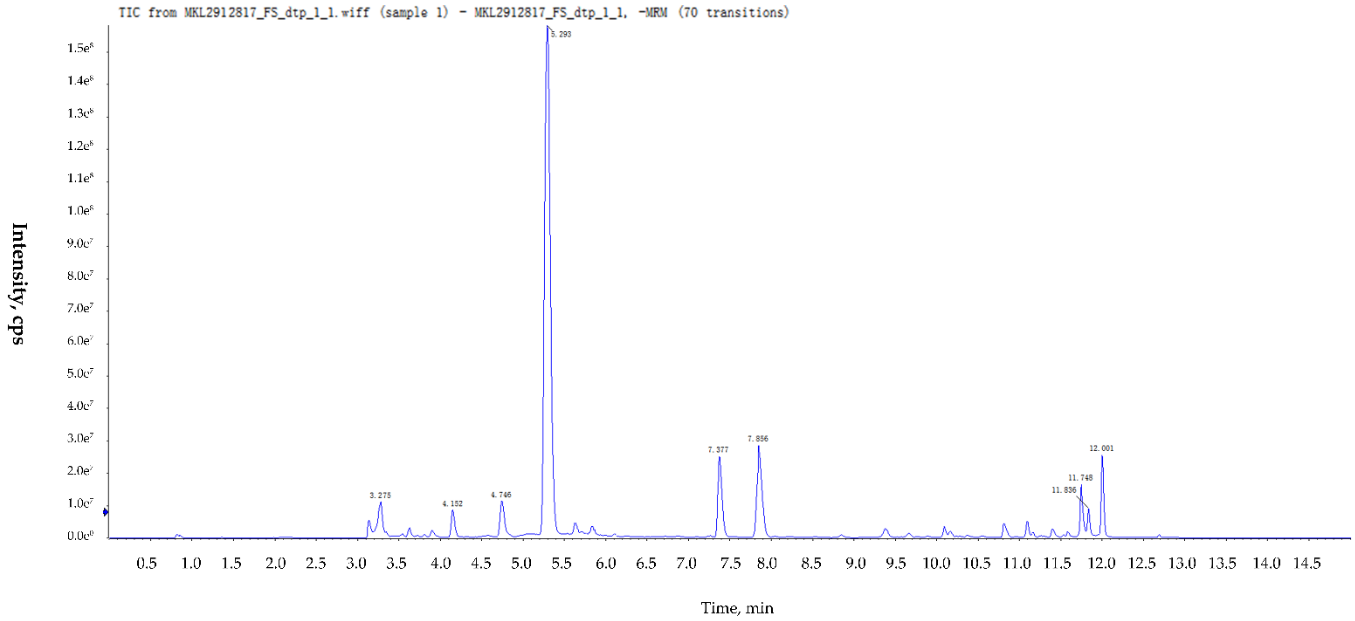

(**b**)

Figure S7. TIC of the Organic Acid Standards and FS_DTP_1: (**a**) TIC of the Organic Acid Standards; (**b**) TIC of one of the samples from FS_DTP_1

Table S1. Proportions of the top 9 fungal genera on wood surfaces in the wood preservation room of Dingtao M2 Tomb (Genus Level, July 2023).

**genus**	**DT1**	**DT2**	**DT3**	**DT4**	**DT5**	**DT6**
Penicillium sp.	36.49%	28.56%	32.24%	0.00%	1.45%	1.30%
Pseudonocardia sp.	4.75%	7.21%	1.54%	1.95%	0.02%	22.25%
Calocera sp.	0.01%	0.02%	0.02%	0.00%	22.00%	0.00%
Dacryopinax sp.	0.00%	0.01%	0.02%	0.00%	18.78%	0.00%
Nocardioides sp.	1.89%	0.07%	0.94%	1.33%	0.01%	11.17%
Mycolicibacterium sp.	0.47%	9.96%	1.18%	1.61%	0.00%	0.67%
Saccharopolyspora sp.	0.06%	0.04%	0.05%	0.05%	0.00%	9.17%
Aspergillus sp.	2.61%	1.68%	7.69%	0.00%	0.05%	0.11%
Kribbella sp.	0.09%	0.04%	0.09%	1.78%	0.00%	7.39%

Table S2. Species α-diversity analysis on wood surfaces in the wood preservation room of Dingtao M2 Tomb(Genus Level，July 2023).

	**DT1**	**DT2**	**DT3**	**DT4**	**DT5**	**DT6**
**Shannon**	3.36	3.61	3.68	6.31	3.07	4.09
**Chao1**	1886.16	1911.25	2139.10	2255.75	1353.29	2220.45
**Goods_coverage**	1.00	1.00	1.00	1.00	1.00	1.00

The Goods_coverage indices of all 6 samples are close to 1.00, indicating a high coverage of the community by the samples, which can represent the species composition of the entire community. The Shannon index of DT4 is highest, suggesting a highest community diversity, characterized by both a greater number of species and a more uniform species distribution. Additionally, its largest Chao1 index indicates that most distinct species are present in the community.

Table S3. Original data and nonlinear fitting data analysis of pH value and growth curve for DTP-1.

	**Time**	**pH**			**Growth curve（mg）**		
**Original data**	0	6.68	6.51	6.53	0.0	0.0	0.0
	12	6.48	6.51	6.46	1.5	1.1	0.9
	24	6.22	6.20	6.23	2.3	1.8	2.2
	36	6.10	6.08	6.10	14.3	15.6	11.1
	48	5.69	5.24	5.56	21.7	22.5	23.2
	60	5.13	5.13	4.76	37.7	27.8	39.3
	72	4.53	4.83	4.85	55.7	44.4	37.7
	84	4.30	4.31	4.32	56.7	50.8	57.5
	96	4.41	4.30	4.30	55.0	58.2	68.7
	108	4.21	3.69	4.09	73.6	80.3	90.1
	120	4.11	4.09	3.18	79.4	81.6	65.8
	132	4.22	4.07	3.24	77.8	92.0	88.3
	144	3.95	3.96	3.40	128.5	105.5	102.6
	156	3.91	4.10	4.00	112.9	103.6	114.9
	168	3.78	4.06	4.06	129.7	132.4	121.0
	180	3.71	3.91	4.11	133.8	129.1	119.9
	192	3.93	4.01	3.91	137.0	111.3	137.6
	204	4.03	4.34	4.24	110.2	125.9	141.5
	216	4.06	4.02	3.62	150.7	142.8	137.0
	228	4.08	3.86	3.73	119.5	152.1	144.8
	240	4.20	4.44	4.07	148.2	138.4	145.6
**Nonlinear fitting data analysis**	YM	3.577			143.2		
	Y0	7.007			6.589		
	k	0.009339			0.02840		
	Xint	107.1			35.21		

Table S4. Table of Information on 67 Organic Acid Standards

Metabolite	CAS ID	Formula	KEGG Compound ID
Taurine	107-35-7	$C_2H_7NO_3S$	C00245
Ethylparaben	120-47-8	$C_9H_{10}O_3$	-
Glycolic acid	79-14-1	$C_2H_4O_3$	C00160;C03547
3-Aminoisobutanoic acid	144-90-1	$C_4H_9NO_2$	C05145
3-Hydroxybutyric acid	300-85-6	$C_4H_8O_3$	C01089
Glyceric acid	473-81-4	$C_3H_6O_4$	C00258
Guanidinoacetic acid	352-97-6	$C_3H_7N_3O_2$	C00581
Isonicotinic acid	55-22-1	$C_6H_5NO_2$	C07446
Nicotinic acid	59-67-6	$C_6H_5NO_2$	C00253
Pipecolinic acid	3105-95-1	$C_6H_{11}NO_2$	C00408
L-Pyroglutamic acid	98-79-3	$C_5H_7NO_3$	C01879
4-Methylbenzoic acid	99-94-5	$C_8H_8O_2$	C01454
4-Hydroxybenzoic acid	99-96-7	$C_7H_6O_3$	C00156
3-Hydroxybenzoic acid	99-6-9	$C_7H_6O_3$	C00587
Salicylic acid	69-72-7	$C_7H_6O_3$	C00805
6-Hydroxynicotinic acid	5006-66-6	$C_6H_5NO_3$	C01020
4-Guanidinobutanoic acid	463-00-3	$C_5H_{11}N_3O_2$	C01035
Cinnamic acid	621-82-9	$C_9H_8O_2$	C10438
Mevalonic acid	150-97-0	$C_6H_{12}O_4$	C00418
Methionine	63-68-3	$C_5H_{11}NO_2S$	C00073;C01733
2-Phenylpropionic acid	492-37-5	$C_9H_{10}O_2$	-
3-Methylsalicylic acid	83-40-9	$C_8H_8O_3$	C14088
4-Hydroxyphenylacetic acid	156-38-7	$C_8H_8O_3$	C00642
3-Hydroxyphenylacetic acid	621-37-4	$C_8H_8O_3$	C05593
5-Aminosalicylic acid	89-57-6	$C_7H_7NO_3$	-
Gentisic acid	490-79-9	$C_7H_6O_4$	C00628
p-Coumaric acid	501-98-4	$C_9H_8O_3$	C00811
3-Hydroxycinnamic acid	14755-02-3	$C_9H_8O_3$	C12621
(S)-2-Hydroxy-3-phenylpropanoic acid	20312-36-1	$C_9H_{10}O_3$	-
Vanillic acid	121-34-6	$C_8H_8O_4$	C06672
Shikimic acid	138-59-0	$C_7H_{10}O_5$	C00493
Hippuric acid	495-69-2	$C_9H_9NO_3$	C01586
Caffeic acid	331-39-5	$C_9H_8O_4$	C01197;C01481
Kynurenic acid	492-27-3	$C_{10}H_7NO_3$	C01717
N-Acetyl-L-methionine	65-82-7	$C_7H_{13}NO_3S$	C02712

2-Methylhippuric acid	42013-20-7	$C_{10}H_{11}NO_3$	C01586
Gluconic acid	527-07-1	$C_6H_{12}O_7$	C00257
Syringic acid	530-57-4	$C_9H_{10}O_5$	C10833
3-Indolebutyric acid	133-32-4	$C_{12}H_{13}NO_2$	C11284
Xanthurenic acid	59-00-7	$C_{10}H_7NO_4$	C02470
Lipoic acid	62-46-4	$C_8H_{14}O_2S_2$	C00725;C16241
Pantothenic acid	79-83-4	$C_9H_{17}NO_5$	C00864
N-Acetyl-L-tyrosine	537-55-3	$C_{11}H_{13}NO_4$	C01657
Oxalic acid	144-62-7	$C_2H_2O_4$	C00209
Malonic acid	141-82-2	$C_3H_4O_4$	C00383;C04025
Fumaric acid	110-17-8	$C_4H_4O_4$	C00122
Methylmalonic acid	516-05-2	$C_4H_6O_4$	C02170
Succinic acid	110-15-6	$C_4H_6O_4$	C00042
Itaconic acid	97-65-4	$C_5H_6O_4$	C00490
Ethylmalonic acid	601-75-2	$C_5H_8O_4$	-
Malic acid	97-67-6	$C_4H_6O_5$	C00149;C00711
D-Tartaric acid	147-71-7	$C_4H_6O_6$	C02107
Chlorogenic acid	327-97-9	$C_{16}H_{18}O_9$	C00852
Phenylpyruvic acid	114-76-1	$C_9H_8O_3$	C00166
2-Isopropylmalic acid	3237-44-3	$C_7H_{12}O_5$	C02504
Azelaic acid	123-99-9	$C_9H_{16}O_4$	C08261
Uridine 5'-monophosphate	58-97-9	$C_9H_{13}N_2O_9P$	C00105
N-Acetyl-L-glutamic acid	1188-37-0	$C_7H_{11}NO_5$	C00624
Sebacic acid	111-20-6	$C_{10}H_{18}O_4$	C08277
Rosmarinic acid	20283-92-5	$C_{18}H_{16}O_8$	C01850
Taurocholic acid	145-42-6	$C_{26}H_{45}NO_7S$	C05122
Cholic acid	81-25-4	$C_{24}H_{40}O_5$	C00695
α-Ketoglutaric acid	328-50-7	$C_5H_6O_5$	C00026
cis-Aconitic acid	585-84-2	$C_6H_6O_6$	C00417
Citric acid	77-92-9	$C_6H_8O_7$	C00158
Isocitric acid	1637-73-6	$C_6H_8O_7$	C00311
Glycocholic acid	475-31-0	$C_{26}H_{43}NO_6$	C01921
